# Supplementary material for: A practical evaluation of correlation filter-based object trackers with new features
Source: PLoS One. 2022 Aug 25;17(8):e0273022. doi: 10.1371/journal.pone.0273022 (PMC9409511; doi:10.1371/journal.pone.0273022)
Supplement: Attachment — Submitted filename: Response to Reviewers.docx [file pone.0273022.s002.docx]

**PLOS ONE**

REPLY TO COMMENTS

**Ref. No.:** PONE-D-21-40546

**Paper Title:** A practical evaluation of correlation filter-based object trackers with new datasets.

Dear Editors and Reviewers,

Thank you for your useful comments and feedback on our paper which helped us to improve its presentation and quality. We have carefully addressed all of your comments in the revised manuscript. We hope that you will be satisfied with the response provided by us.

Sincerely,

The authors.

**Journal Requirements**

**Comment 1:** Please ensure that your manuscript meets PLOS ONE's style requirements, including those for file naming.

**Response 1:** We followed the indicated instructions.

**Comment 2:** PLOS requires an ORCID iD for the corresponding author in Editorial Manager on papers submitted after December 6th, 2016. Please ensure that you have an ORCID iD and that it is validated in Editorial Manager.

**Response 2:** We followed the indicated instructions.

**Comment 3:** We note that Figures 4, 6, 7 in your submission contain copyrighted images. All PLOS content is published under the Creative Commons Attribution License (CC BY 4.0), which means that the manuscript, images, and Supporting Information files will be freely available online, and any third party is permitted to access, download, copy, distribute, and use these materials in any way, even commercially, with proper attribution.

**Response 3:** Object Tracking Benchmark (**OTB**) is a visual tracking benchmark that is widely used to evaluate the performance of a visual tracking algorithm. There are tens of papers using images of these dataset. In addition, the images of Figs. 4, 6, and 7 are modified images, as the original images don’t have trackers’ boundary boxes.

**Reviewer #1’s comments**

There are clear weaknesses in the paper that the authors must particularly pay attention and handle:

**Comment 1.1:** The major problem of this work is that its novelty is very limited where some methods have already been used in the literature. In addition, the theoretical contribution is so limited. So, the authors should modify it carefully and improve the novelty of this paper. Also, the authors should provide solid motivation for their work based on the existing literature.
**Response 1.1:** Authors are thankful to the reviewer for this constructive suggestion. In the revised manuscript, we provided **solid motivations** as follows:

1) The lack of practical evaluation of the recent CF-based object trackers motivated this work as well.
2) The existing standard video sequences used for the practical evaluation of the object trackers contain objects that are easy to be tracked by human beings. Thus, there is a need to evaluate object trackers with a video sequence that is hard to follow by a human.

The aim of this manuscript is to practically evaluate the recent CF-based trackers. Thus, we focused on the practical side of the trackers in the initial manuscript. As suggested, **the theoretical contribution** is extended by discussing the merits and demerits of the recent CF-based trackers in details. In addition, the performance of the trackers are justified, as explained in Section 2.2 of the revised manuscript.

Finally, the novelty of this work can be summarized as follows:

1) We proposed a set of new video sequences with new attributes, namely, hard-to-follow-by-human and very fast motion. To our knowledge, it is the first
   time to propose evaluating object trackers with a video sequence which is hard to
   follow by human’s eyes.
2) We thoroughly practically evaluated eight recent CF-based trackers through a set
   of experiments, and the obtained results outlined the merits and demerits of these
   eight trackers.

**Comment 1.2:** The first occurrence of abbreviations of technical terms should be explained with the full name. For example, "LDES" in Abstract.”
**Response 1.2:** Authors are thankful to the reviewer for this constructive suggestion. In the revised manuscript, we provided the full name of each abbreviation for the first occurrence, as suggested.

**Comment 1.3:** Figures. 9 and 10 need to be amended, where the font in figures is slightly small to be seen which make them difficult to read.

**Response 1.3:** Authors are thankful to the reviewer for this constructive suggestion. In the revised manuscript, the font size of these two figures increased to readable font size.

**Comment 1.4:** The review for most of the cited work is nebulous. The review failed to summarize critical details about each of the cited research study and its relevant pros and cons.

**Response 1.4:** Authors are thankful to the reviewer for this critical suggestion. In the revised manuscript, the cited researches are exposed in better way to outline the basic idea, advantages and disadvantages of each method, as exposed in Section 2.2.

**Comment 1.5:** Section 3 and 4 need more explanations and details which are the core of this paper.

**Response 1.5:** Authors are thankful to the reviewer for this critical suggestion. In the revised manuscript, Sections 3 and 4 are swapped. In the revised manuscript, Section 3 “Dataset Formulation” is exposed in a very detailed manner, as dataset formulation is a major contribution of this work. Section 4 “Evaluation Metrics” explains well known performance metrics, which is not our contribution. Thus, we didn’t extend Section 4 “Evaluation Metrics”.

**Comment 1.6:** The results should be further analyzed, more details and further discussion of the simulation results is needed.
**Response 1.6:** Authors are thankful to the reviewer for this constructive suggestion. In the revised manuscript, we exposed the results in more detail.

**Comment 1.7:** The conclusions section should conclude that you have achieved from the study, contributions of the study to academics and practices. In addition, list the advantages and disadvantages of the proposed solution, as well as indicate the limitations of work. Further, mention the recommendations of future works.

**Response 1.7:** Authors are thankful to the reviewer for this critical suggestion. In the revised manuscript, we rewritten the conclusion section to include all the suggested points.

**Comment 1.8:** The writing of the paper needs work. It is full of syntactical and grammatical mistakes, that make its understandability extremely difficult. A thorough proofreading is required (best by a native English speaker).
**Response 1.8:** Authors are thankful to the reviewer for this critical suggestion. As suggested, the manuscript was proofread by a native English speaker.

**Comment 1.9:** The list of references should be reformatted and checked again to be matched with the journal requirement where a different styles and types are used. Please check some spells and typos.

**Response 1.9:** Authors are thankful to the reviewer for this critical suggestion. As suggested, the references are checked properly.

**Comment 1.10:** In addition, I recommend the authors to read and cite the following paper which is recently done and will be helpful to the revision of this paper:
1. " A background-aware correlation filter with adaptive saliency-aware regularization for visual tracking.", 2022.
2. " Liu, Bing, et al. "HCDC-SRCF tracker: Learning an adaptively multi-feature fuse tracker in spatial regularized
correlation filters framework.", 2022.
3. "Accelerated duality-aware correlation filters for visual tracking." Neural Computing and Applications.” 2022.
4. " Visual tracking: Tracking in scenes containing multiple moving objects.” 2022.

**Response 1.10:** Authors are thankful to the reviewer for this suggestion. As suggested, all of these references are discussed and cited.

**Reviewer #2’s Comments**

The paper proposed a set of new sequences with higher difficulties than the existing object tracking benchmarking sequences, and evaluated eight CF-based trackers through a set of experiments.

**Comment 2.1:** The paper introduced basic concepts such as simple correlation and general correlation filter-based trackers very well, however, recent research (papers in recent one or two years) may be overlooked.
**Response 2.1:** Authors are thankful to the reviewer for this suggestion. As suggested, the revised manuscript discussed eight papers in the last two years, 2021 and 2022.

**Comment 2.2:** The three main contributions claimed in Introduction were not supported sufficiently by the following sections.
**Response 2.2:** Authors are thankful to the reviewer for this critical suggestion. As suggested, the list of contributions improved and explained in more details, as detailed in the “Introduction”, “Related Work”, and “Data Formulation” sections.

In addition, here is some other advice.
**Comment 2.3:** It is required to give the full form of abbreviations when they first appear. Eg., abbreviations on P2.
**Response 2.3:** Authors are thankful to the reviewer for this constructive suggestion. In the revised manuscript, we provided the full name of each abbreviation for the first occurrence, as suggested.

**Comment 2.4:** What does it mean by "The two images in Fig. 3…" in the last paragraph on P3?
**Response 2.4:** Authors are thankful to the reviewer for this constructive suggestion. We apologize for this mistake. We explained Fig. 3 in a better way to remove this ambiguous meaning, as explained in the first paragraph on page 5. We added the following sentences:

“Fig. 3 includes one large image and one small sub-image. The small sub-image represent the patter, i.e., the object to be tracked, and the large image represents the image for testing, an image with many patterns. The small sub-image appears at the top-left corner of the large image; it is the image with kiwi fruit.”

**Comment 2.5:** Differences between OTB original sequences and the modified sequences can not be shown clearly on Fig. 4. It would be better to present another better expression method.
**Response 2.5:** Authors are thankful to the reviewer for this constructive suggestion. We agree with the reviewer on this concern. We removed this figure from the manuscript and explain in detail the difference between the original and new video sequences in Section 3. In addition, we uploaded sample sequences in the supplementary material section of this paper.

**Comment 2.6:** What are main differences between microscopic video sequences and macroscopic video sequences? Since it was claimed to be proposed for the first time, it would be better to pay more attention to depict and discuss about these microscopic sequences.

**Response 2.6:** Authors are thankful to the reviewer for this constructive suggestion. The macroscopic level includes anything the human being can see with the naked eye and the microscopic level includes things not seen with the human being naked eye. In this work, we proposed tracking the Amoeba movement, which is at the microscopic level. The justifications for using such a sequence are detailed in Section 3.

**Comment 2.7:** It may be regarded as a main contribution to propose a set of new sequences, then it would be better to explain the idea and processes more detailed, such as why to choose these criteria, how to highlight the innovation, etc. Especially, how significant are differences between OTB original sequences and the modified sequences? How to measure the significance of differences?

**Response 2.7:** Authors are thankful to the reviewer for this constructive suggestion. As suggested, in Section 3 of the revised manuscript, we detailed the significance and intuition of the new and modified OTB-2013 sequences.

**Comment 2.8:** After describing experiment results, it is supposed to present some deep discussion. Why does one tracker outperform others? Otherwise, the contribution is not persuasive.

**Response 2.8:** Authors are thankful to the reviewer for this constructive suggestion. In the revised manuscript, we exposed the results in more detail.

**Comment 2.9:** It would be better to further refine the innovation and contribution.

**Response 2.9:** Authors are thankful to the reviewer for this constructive suggestion. In the revised manuscript, we exposed these two issues in a better way as follows.

The aim of this manuscript is to practically evaluate the recent CF-based trackers. Thus, we focused on the practical side of the trackers in the initial manuscript. As suggested, **the theoretical contribution** is extended by discussing the merits and demerits of the recent CF-based trackers in details. In addition, the performance of the trackers are justified, as explained in Section 2.2 of the revised manuscript.

Finally, the novelty of this work can be summarized as follows:

3) We proposed a set of new video sequences with new attributes, namely, hard-to-follow-by-human and very fast motion. To our knowledge, it is the first
   time to propose evaluating object trackers with a video sequence which is hard to
   follow by human’s eyes.
4) We thoroughly practically evaluated eight recent CF-based trackers through a set
   of experiments, and the obtained results outlined the merits and demerits of these
   eight trackers.
